# The infant microbiota hopscotches between community states toward maturation—longitudinal stool parameters and microbiota development in a cohort of European toddlers

**DOI:** 10.1093/ismeco/ycaf016

**Published:** 2025-03-11

**Authors:** Evangelia Intze, Monika Schaubeck, Mohsen Pourjam, Klaus Neuhaus, Ilias Lagkouvardos, Thomas C A Hitch, Thomas Clavel

**Affiliations:** Department of Clinical Microbiology, School of Medicine, University of Crete, Heraklion 70013, Greece; HiPP GmbH and Co. Vertrieb KG, 85276 Pfaffenhofen, Germany; Core Facility Microbiome, ZIEL Institute for Food & Health, Technical University of Munich, 85354 Freising, Germany; Core Facility Microbiome, ZIEL Institute for Food & Health, Technical University of Munich, 85354 Freising, Germany; Department of Clinical Microbiology, School of Medicine, University of Crete, Heraklion 70013, Greece; Functional Microbiome Research Group, Institute of Medical Microbiology, RWTH University Hospital, 52074 Aachen, Germany; Functional Microbiome Research Group, Institute of Medical Microbiology, RWTH University Hospital, 52074 Aachen, Germany; Functional Microbiome Research Group, Institute of Medical Microbiology, RWTH University Hospital, 52074 Aachen, Germany

**Keywords:** infant gut microbiota, microbiome development, community states, early life, double-blinded controlled study, infant formula, synbiotics

## Abstract

The development of the gut microbiome is critical during early life and is associated with infant health. To test whether this development is deterministic and how it is influenced by factors such as diet and mode of birth, we studied microbiota profiles and fecal parameters of 540 European infants, fed a synbiotic or control infant formula during their first year of life, up to 36 months of age. The diversity of the microbiota gradually increased until 36 months, at which point it resembled adult community states, indicating that microbiota maturation had occurred. However, distinct gut microbiota community states were observed that differed at each stage of maturation. The distribution of infants within the communities even at 36 months was significantly influenced by early life events, with a higher prevalence of infants born by cesarean section having the immature M36-C1 community state at 36 months. The microbial community state at one time point was not predictive of the next; instead, we observed hopscotching of the infant microbiota between different community states. This work provides new longitudinal data on the infant gut microbiome in relation to diet, suggesting that ecosystem development is not deterministic, but that early life events influence the community state of an individual's gut microbiota beyond infancy.

## Graphical Abstract

**Graphical Abstract:**
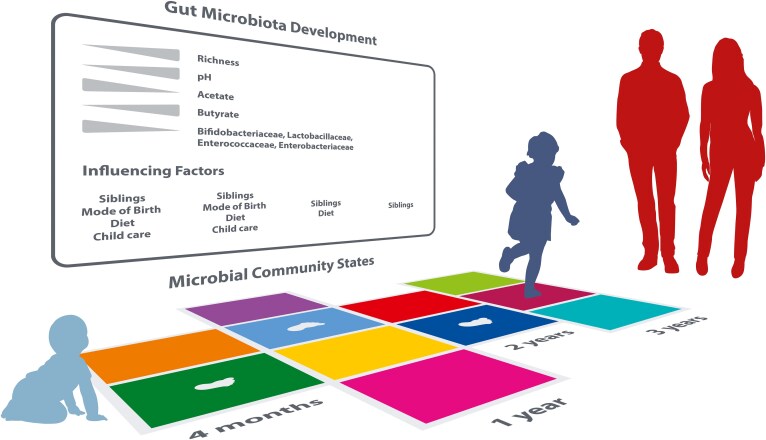

## Introduction

The first months of life have a lasting impact on the development of the infant’s immune system, termed the neonatal window of opportunity [1]. This coincides with the rapid development of body-associated microbial communities [2]. The maturation of the gut microbiota during early life is of great interest, as it may be associated with the risk of pathologies in later life [3, 4].

Previous research has suggested that the infant gut microbiota can be separated into distinct communities [5] and that the development of the infant gut microbiota, to adulthood, follows a deterministic progression through community states [6]. While the general trend of gut microbiota development toward a mature state is deterministic, the transition between community states that a given individual’s microbiota will undergo appears to be chaotic [6–8]. One of the most important factors that influence microbiota development is diet. Several studies have shown the impact of early nutrition on infant microbiome development [9, 10]. While formula-fed infants have historically been grouped together, formula diets vary widely in their composition and ingredients used. These include probiotic bacterial species and prebiotic carbohydrates [11], such as galacto-oligosaccharides (GOS) that support the growth of bifidobacteria and contribute to soft stools and low fecal pH in infants [12, 13]. In our previous work, we showed that diet, specifically a comparison between infant formula with or without synbiotic (*Limosilactobacillus fermentum* CECT5716 and GOS) and a reference group fed only human milk (HM), had the greatest effect on microbiota composition at 4 months of age compared with sampling at 12 and 24 months [14]. As few studies have looked into the factors that determine microbiota development at an age older than 24 months [2, 8], cohorts with longitudinal sampling and collection of external parameters that may influence microbiota development are needed. The lack of longitudinal data, or metadata, may prevent the development of an individual’s microbiota from being investigated, and potentially predicted from early states as suggested by previous studies [6].

The aim of this study was to refine our understanding of developmental trajectories of the infant gut microbiota during the first 3 years of life (4, 12, 24, and 36 months), both to describe the community states and to quantify the potential to predict the development of a microbiota through these community states. This was achieved using 16S ribosomal ribonucleic acid (rRNA) gene amplicon sequencing and fecal parameters from a multicenter cohort of 540 infants (1353 samples in total). The effect of early life events, such as mode of delivery and diet, on community assembly and predicted functional capacity was determined up to the age of 3 years. The inclusion of fecal mature microbiota (MM) profiles from a cohort of 216 healthy adults throughout the analysis allowed the study of the trajectory of the infant gut microbiota toward a mature state.

## Materials and methods

### Study design

Between August 2014 and May 2018, healthy infants (*n* = 540) from either France or Belgium (40 sites total) were included in a double-blinded, controlled trial. Infants whose parents had chosen not to breastfeed or were not able to breastfeed prior to study inclusion were allocated randomly to 1 of 2 formula groups (CF, control formula without synbiotic, *n* = 230; IF, intervention formula with synbiotic, *n* = 230). The infants in the breastfed reference group (*n* = 80) were fed mainly with HM, i.e. with a maximum of 1 formula meal per 24 h and willingness of the mother to continue breastfeeding at least until the age of 4 months. Further details on exclusion criteria and power calculations are detailed in our previous publication [14]. In brief, infants were excluded in case of history of neonatal health problems, clinical evidence of chronic illnesses or gastrointestinal disorders, diagnosed metabolic or immune disorder, consumption of formula for special medical purposes, or oral antibiotic treatment at the time of enrolment.

IF was a standard infant formula enriched with prebiotic GOS (0.02 g/g) and the probiotic strain *Lactobacillus fermentum* CECT 5716 (at least 1.0 × 10^6^ colony forming units (cfu)/g resulting in an average daily dose at least 2.0 × 10^8^ cfu/d), creating a synbiotic formula. The follow-on formula was a standard follow-on formula enriched with 0.03 g/g GOS and at least 1.5 × 10^6^ cfu/g (to compensate for reduced drinking volumes in this age group). CF had similar constituents apart from the pre- and probiotics. All study products were in accordance with EC Directive 2006/141/EC. Formula consumption was *ad libitum*, starting within the first 3 days after inclusion (1 month ± 7 days) until 6 months of age, followed by follow-on formula until 12 months of age. At 4 months, the drinking volume in both formula groups was approximately 780 ml.

This clinical trial was prospectively registered in clinicaltrials.gov under the name “The Combiotic-Study (GOLFIII)” (NCT02221687) and carried out in accordance with the Declaration of Helsinki and the Good Clinical Practice standards as they apply to nutritional trials. Ethical approval for the study was obtained from the Ethics Committees Ouest IV of Nantes, France, and University Hospital Saint-Luc, Belgium (Nr. 15/14 from 1 April 2014). Parents of all included infants provided written informed consent for study participation.

### Infant sample collection

Stool samples were collected by the parents directly from diapers as soon as possible after defecation. As described previously, this was done once at 4, 12 and 24 months of age [14]. In this work, we additionally analysed samples from 36 months of age. Once collected and transported to the laboratories, stool samples were transferred into 2 microtubes, 1 for microbiota analysis by sequencing, and 1 for additional measurements: calprotectin, secretory immunoglobulin A (sIgA), pH, fecal water, and short-chain fatty acids (SCFAs). Microtubes were stored at −20°C until being further analysed, no later than 6 months after sample collection. As not all participants provided stool samples at the study visit time points, the number of analysed stool samples per time point were as follows: M4: 419; M12: 379; M24: 289; M36: 257.

### Fecal parameters

The pH of the stool samples was measured with a pH meter (pH 1000 L, VWR International). Prior to SCFA analysis, the humidity rate of the stool samples was determined using an oven (Memmert GmbH + Co.KG) at 105 ± 5°C for 24 h. Weight difference before and after drying due to water loss was assessed and the humidity rate was calculated in percentage afterwards. To measure SCFAs, stool samples were homogenized, dissolved in water and ultrasonicated before centrifugation. SCFAs were extracted with ethyl ether after adding hydrochloric acid solution and the internal standard. A fraction of the organic phase was dried with sodium sulfate and introduced into a vial for gas chromatography. The samples and the calibration range were analysed by gas chromatography with flame ionization detection (Shimadzu GC-2010 Plus) using a 30 m × 0.25 mm × 0.25 μm J&W Scientific capillary column. The obtained SCFA results were expressed as mg/g dry fecal matter. sIgA concentration was determined by ELISA (ImmuChrom ELISA Kit, ImmuChrom GmbH) according to the manufacturer’s instructions. Calprotectin concentration was measured by ELISA or turbidimetric assay (Bühlmann fCAL tests) according to the manufacturer’s instructions.

### Reference adult samples

Stool samples from 216 healthy young adults (mean BMI, 21.65; mean age, 23) from the School of Life Sciences Weihenstephan of the Technical University of Munich were collected anonymously. Ethical approval for the study was obtained from the Ethics Committees of the Technical University of Munich (No. 2024-5-NM-KH). Based on their answers to a detailed questionnaire, none of the donors had been taking antibiotics in the past 3 months, had any illness or had been taking long-term medication as described in detail in [15].

### Sequencing

Extraction of deoxyribonucleic acid (DNA) from stool samples was conducted using the Maxwell 16 Tissue DNA Purification kit (Promega) with a dual cell lysis protocol (mechanical and chemical) as previously described [14]. This was conducted on a Maxwell MDx automated station (Promega). A Qubit 2.0 fluorometer (Thermo Fisher Scientific) quantified the DNA. Polymerase chain reaction using the primers 341F and 785R amplified the V3-V4 regions of the genes encoding 16S rRNA as previously described [14]. A 16S rRNA gene amplicon library was generated for each sample by adding dual indices and Illumina sequencing adapters using the Nextera XT Index kit. Each library was cleaned up with magnetic AMPure XP beads (Beckman Coulter) and the size was verified for at least 10% of the samples by capillary electrophoresis with a 2100 Bioanalyzer (Agilent Technologies). Libraries were normalized to 4 nM and pooled before denaturation and sequencing (paired-end, 2 × 250, v2 chemistry) using an Illumina MiSeq.

### Analysis

The raw FASTQ files were processed through the IMNGS platform [16], implementing the UNOISE3 [17] algorithm from the USEARCH11 [18] package, using the default parameters. The produced denoised sequences were clustered at 97% sequence similarity using the UPARSE algorithm [19] and then were aligned and classified by SINA aligner [20] using as reference the SILVA release 138 [21]. The generated sequences' taxonomic assignments were manually refined and a phylogenetic tree was calculated with the Neighbor-Joining method in MEGAX [22].

A total of 127 million high-quality V3–4 amplicon sequences were processed (119.5 million for infants; 7.5 million for adults), with an average sequencing depth of 88 600 ± 45 000 (infants) and 32 100 ± 20 000 (adults) reads per sample. This provided a total diversity of 629 sOTUs (117 ± 40, infants; 144 ± 35, adults). While the samples from the time points M4, M12 and M24 were already included in our previous analysis [14], we included samples from 257 infants at the age of 36 months and 216 samples from an adult reference cohort to investigate factors influencing the infant microbiome maturation toward toddler age. In total, 119 samples did not fulfill the quality and criterion assessment and were therefore excluded from the analysis. The downstream analysis was performed in R programming language (version 4.3.0) using the Rhea pipeline (version 1.1.6) [23]. For the α- and β-diversity analysis, the raw counts were normalized so that the sum of their counts was equal across all the samples. Thus, the raw counts were normalized using Total Sum Scaling, scaled down to the size of the sample with the smallest number of reads (11059).

The α-diversity was measured in terms of richness and effective richness [24], while the β-diversity was calculated using generalized Unifrac [25], setting the parameter α = 0.5 and visualizing the results through the form of MDS plots. For the taxa composition comparisons, the raw counts were transformed into their relative abundances and then compared against each other. The distances-based part of the analysis and the comparisons between the reference and test groups were performed using DivCom [26]. The transition trajectories of the samples over time and the model-based evaluation of them were performed using Cronos [27]. The groups were clustered using the Partition Around Medoids (PAM) algorithm and the dissimilarity matrix as it was calculated by the generalized Unifrac (α = 0.5). The optimal number of clusters for each group was assessed by the Calinski–Harabasz index. The multinomial logistic regression algorithm was used to estimate the accuracies of the clusters transition model.

The predicted functional abundance analysis of the samples was performed using PICRUSt2 [28]. The script “picrust2_pipeline.py” produced the necessary tables and then the script “add_descriptions.py” added the descriptions to them. The produced ECs and MetaCyc-annotated pathway tables were further analysed using Rhea and a modified version of DivCom. Functional distances between the samples were calculated using the Bray-Curtis metric. For all of the aforementioned pipelines, their default parameters were used.

The differences in β-diversity were evaluated using the permutational multivariate analysis of variance (PERMANOVA) [29], while the homogeneity of the groups was assessed using the permutational analysis of multivariate dispersions [30]. Both tests were performed using functions provided by the vegan package in R. The differences in the relative abundances of taxa between the groups were assessed using the Kruskal-Wallis rank sum test and the differences in their prevalence were tested using Fisher’s exact test. In the cases where the adjusted p-value of these tests reached the cutoff of 0.05, pairwise comparisons were followed using the Wilcoxon rank sum test and Fisher’s exact test.

Besides the taxa comparison between the groups, the same approach was used for the assessment of the statistically significant pathways among the groups. The pairwise comparisons of the sample distributions across the groups were tested with the Chi-square goodness of fit test. All the p-values were adjusted using the Benjamini–Hochberg method and adjusted p-values under the cut-off of 0.05 were considered statistically significant.

## Results

### Sequential maturation of the infant gut toward a mature microbiota

**Figure 1:**
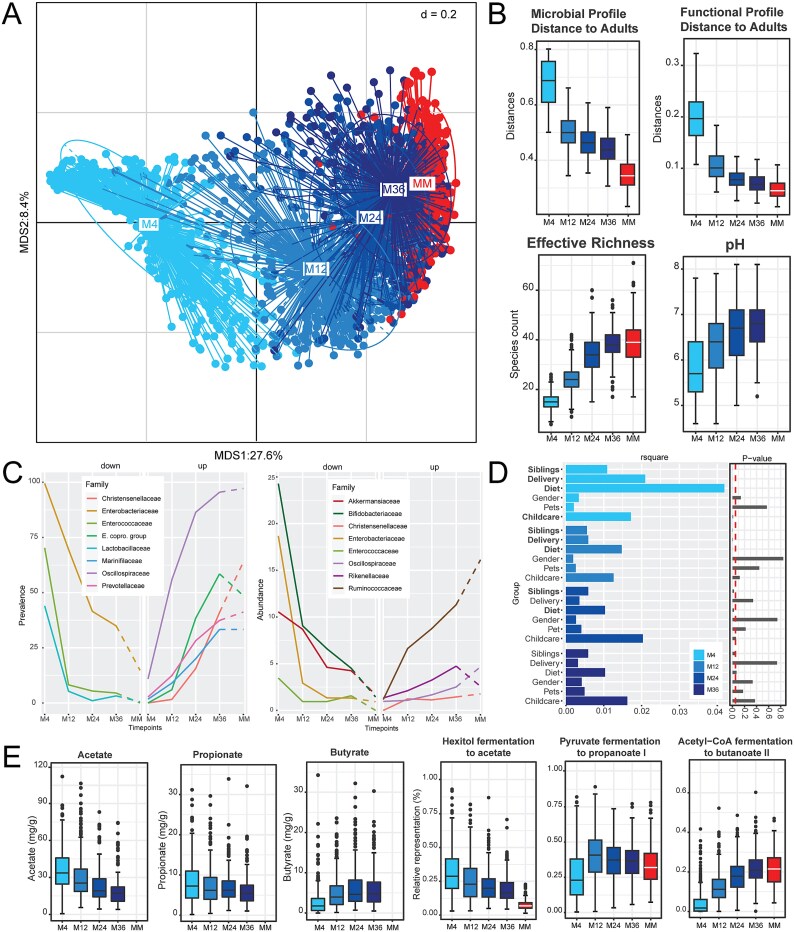
Development of the infant microbiome over the first three years of life. A. MDS plot of all samples (*n* = 1569) over time using generalized Unifrac distances. B. Phylogenetic distances of infants’ microbial profiles relative to the reference MM samples (top left plot). Distances (Bray–Curtis) of the infants’ predicted ECs profiles relative to the reference MM samples profiles (top right; PICRUSt2 data). Effective richness [24] within the samples of each time point (bottom left). Measured pH of the fecal samples; pH was not measured in MM samples (bottom right). C. Mean prevalence (left) and relative abundance (right) of dominant bacterial families over time that displayed a monotonic pattern of increasing or decreasing toward MM samples. The dashed lines show the corresponding value of the MM samples (unpaired samples). E. Copro group is an abbreviation for the taxonomic group within the SILVA database termed “[Eubacterium] coprostanoligenes group”. D. Explained variance (R-square values) of the variables as determined by permutation PERMANOVA along with their p-value for each time point. Factors identified to have a significant (*P* < .05) effect on the microbiota at the studied time point are written with bold letters. Diet refers to each infant’s dietary grouping during the first 12 months of life (i.e., CF or IF group; the HM reference group was not randomized), as detailed in the study design. E. Acetate, propionate, and butyrate concentrations in stool (left panels) and their associated predicted pathways (right panels). In the PICRUSt2 analysis, the relative representation (Y-axis, in %) displays the predicted microbiome fraction associated with the given functional pathway. Abbreviations: M4, 4-month-old infants; M12, 12-month-old infants; M24, 24-month-old infants; M36, 36-month-old infants; Statistical significance values for all plots are provided in Supplementary Table 1.

Over the first three years of life, the infant microbiota profiles converged towards a mature microbiota (Fig. 1A). The most dramatic shift towards the MM samples, both phylogenetically and in terms of functional prediction (PICRUSt2), occurred between 4 and 12 months (Fig. 1B). At 24 and 36 months, the distance to the MM samples was almost comparable (Fig. 1B top). This plateau was somewhat reflected in the effective richness of the samples, with the infant gut at 36 months showing the same richness as the MM samples (Fig. 1B bottom). An increase in pH over time occurred that followed a similar pattern to the increase in richness; the correlation between these factors was minor, but significant (Rho = 0.37; *P* = .001). The changes in richness were reflected by substantial changes in the prevalence and relative abundance of several dominant bacterial families (Fig. 1C). Additional parameters (pH, sIgA, Calprotectin, humidity) were measured in all infant fecal samples and correlations with the occurrence of taxonomic groups were calculated, but no strong associations were identified at each time point (Supplementary Table 1).

While these changes occurred sequentially, based on age, the variation of bacterial relative abundances between infants at each time point was large, suggesting that external factors have an influence. Based on the available metadata for each infant, we used PERMANOVA analysis to quantify the impact of external factors (i.e. presence of siblings in the household, mode of birth, diet in the first 12 months of life, gender, pets, and attendance at childcare facilities) on the microbiota at each time point (Fig. 1D). The infants within this study were in one of three feeding groups, in which the infants received either exclusively HM or were provided one of two formulas (IF, with synbiotic; CF, without synbiotic) for at least the first 12 months. We observed that the infants diet during the intervention period had a statistically significant impact until 2 years of age (*P* = .025). However, this influence was by far the highest at the age of 4 months, with a substantial lower impact already at 12 months. The presence of older siblings within the home also had a significantly long-lasting impact on the infant microbiome, remaining significant until 2 years (*P* = .025).

The changes in microbiota composition were paralleled by changes in fecal SCFAs (Fig. 1E). The lowest butyrate values were observed at M4, which then increased sequentially until M24. In contrast, lower microbial acetate and propionate production were observed by the second year of life (M24 and M36), compared to the first year of life. As SCFAs were not measured in the MM samples, PICRUSt2 was used for functional prediction based on 16S rRNA gene amplicon profiles. The pathways relating to each of the measured SCFAs reflected the same patterns.

### Diet is most impactful on the microbiota during the first 4 months of life

**Figure 2:**
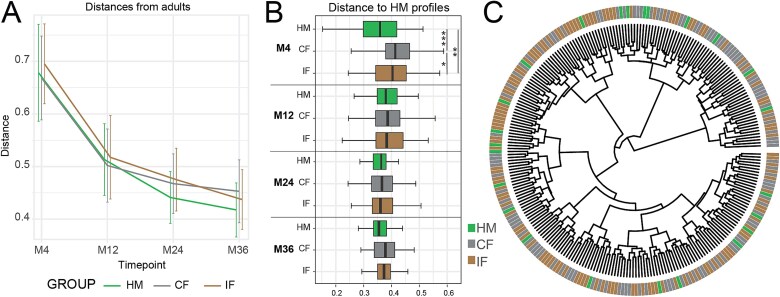
Influence of early diet on the microbiome in the first 3 years of life. A. The distance of feeding groups from the MM samples for each time point are shown at the level of microbial composition (generalized Unifrac distance). B. Boxplots of the distances between the diet groups (CF and IF) and the HM group within each time point via microbial composition. C. Cladogram of individual microbial profiles based on generalized Unifrac distances. IF (with synbiotic; M4, 4-month-old infants; M12, 12-month-old infants; M24, 24-month-old infants; M36, 36-month-old infants). Significance differences for all plots can be found in Supplementary Table 1.

We previously observed that the microbiota of infants fed IF shared more similarities with the microbiota of infants fed HM compared to the CF, especially when the infants were born by cesarean section (CS) [14]. In the present study, we explored this impact further until 36 months of age and in relation to the mature microbial profiles of an adult cohort. We observed that all three diet groups displayed the same trajectory of maturation toward profiles in the MM samples (Fig. 2A). At M36, the compositional profile of infants fed CF was significantly different from the HM group (*P* = .01), while no significance was found between the IF and HM infants (*P* = .147), although these diet differences were marginal compared to the changes over time (Fig. 2A). Using the HM-fed infants as a reference group, we assessed the distance of both formula-fed infant groups over time (Fig. 2B). While significant differences were observed between both formulas and the HM group at 4 months, no differences were observed after this time point. Interestingly, at the age of 4 months we observed a significant difference between the two formulas with the IF group showing a slightly reduced distance to the HM group compared to CF. At 36 months of age, we observed that in comparison to both, the MM samples and to each other (i.e., IF *vs*. CF), the dietary groups showed minor differences taxonomically. To validate this further, dendrograms of the similarity of all 36-months samples to each other were generated using the taxonomic distances, showing no clear clustering based on the early dietary interventions (Fig. 2C). These results suggest that diet in the first 6 months of life influences the microbiota during this period; however, the long-term impact of these changes on the microbiota, as detected by amplicon sequencing are minimal. These observations were confirmed at the level of inferred predicted functional profiles (Supplementary Information, Supplementary Figure S1 and Supplementary Table 2).

### Community states development over the first 3 years of life

**Figure 3:**
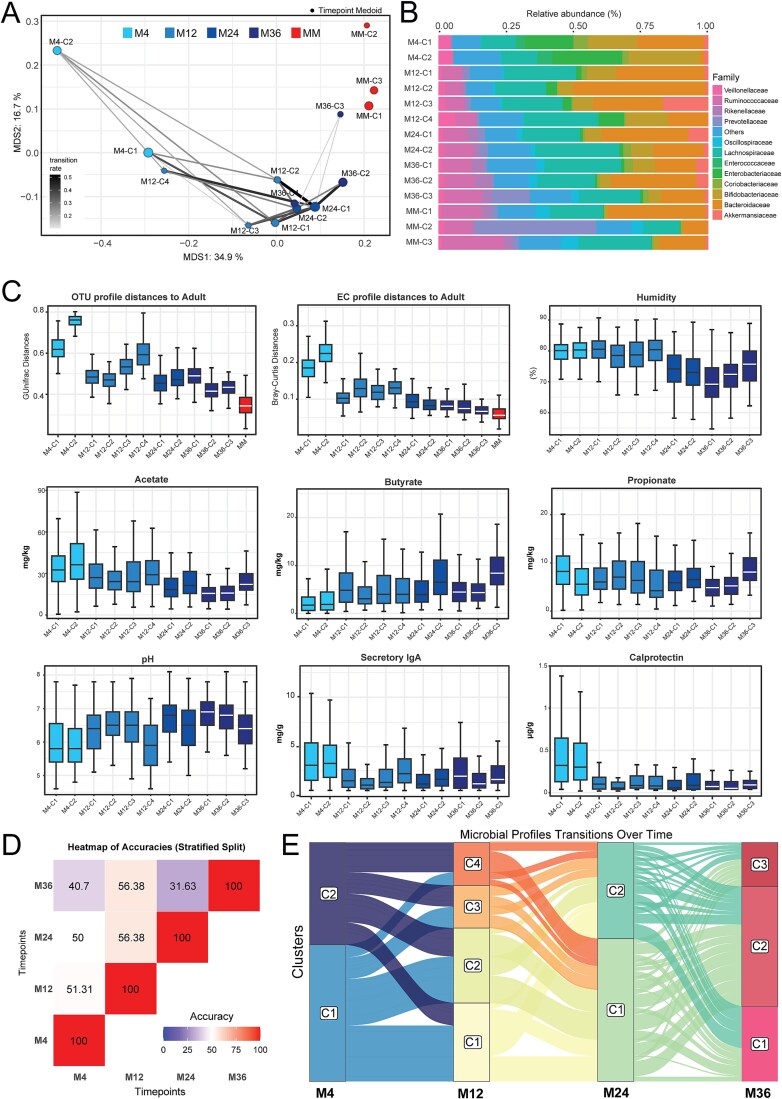
Development of the infant microbiota: A hopscotch between age-specific community states. A. MDS plot presenting the relative position of microbiota community states; the thickness of lines represents the proportion of infants that transitioned between the linked community states; the size of each community states dot is proportional to the percentage of samples from that time point assigned to the specific community state (size range: 16.6%–58% of infants). B. Average taxonomic composition of each microbiota community state at the family level. C. Distances between the microbial profiles of all infant samples that belonged to each microbiota community state and those from the reference MM samples based on phylogeny (generalized Unifrac; first plot) or functions (Bray–Curtis on predicted ECs; second plot). The remaining plots display measured parameters of the fecal samples: humidity, pH, acetate, butyrate, propionate, sIgA, and calprotectin. D. Prediction accuracy of multinomial logistic regression models for success in predicting the current community state of infant microbiota and their future community state E. Alluvial diagram of the dynamics of sample-allocation to the different microbiota community states over time. Abbreviations: C, community state; M, month; M4, 4-month-old infants; M12, 12-month-old infants; M24, 24-month-old infants; M36, 36-month-old infants. Significance differences for all plots can be found in Supplementary Table 1.

With increasing age, a general pattern of microbiota maturation toward MM samples is observable. However, the impact of external influences (diet, siblings, etc.) on the microbiota may lead to variation in community states at each time point during maturation. To assess this, we determined the optimal number of community states for each time point using the Calinski–Harabasz index and then performed unsupervised clustering, leading to the recovery of 2–4 community states at each time point (Fig. 3A). When studying the progression of samples through these age-specific community states, we observed a number of paths that an individual can transition through, with no single transition between community states accounting for more than 50% of samples. The key differences between each community state were identified on the basis of their taxonomic profiles (Fig. 3B). At 4 months, two community states were identified: one state (M4-C1) showed high relative abundance of *Bacteroidaceae* (24.8 ± 13.9%), while the other (M4-C2) was dominated by *Bifidobacteriaceae* (26.2 ± 14.1% relative abundance), as reported previously [14]. The greatest number of community states was observed at 12 months, with four states. Between these community states, we identified multiple taxonomic families which appeared to contribute toward their separation. *Bifidobacteriaceae* and *Bacteroidaceae* were observed to significantly differ in relative abundance between all four community states. In all of them, *Akkermansiaceae* was detectable, but a relative abundance greater than 2% was only displayed in state M12-C3 (16.9 ± 10.0%). *Enterobacteriaceae* was also detected within all four community states, with highest relative abundance in M12-C4 (9.3 ± 1.4%). By 36 months, the separation of community states was driven by multiple families rather than a single dominant family.

By comparing the infant community states with the MM samples, we were able to infer the level of maturation per state during the first 3 years, both taxonomically and functionally-predicted (Fig. 3C). Overall, the distance to the MM samples of all community states at a given time point decreased with age. The variation in operational taxonomic unit (OTU) profile distance and predicted enzyme commission number (EC) profile distance to adults (first two boxplots panels in Fig. 3C) was reflected in the measured fecal parameters (all other boxplots in Fig. 3C). Humidity, a proxy for fecal water content, was most variable at 36 months, with M36-C1 having the lowest humidity (70.4 ± 7%) compared to M36-C3 (74 ± 9%; *P* = .0005), which coincides with M36-C1 having a significantly greater distance to the MM samples than either of the other community states at 36 months (Supplementary Table 1). Similarly, M12-C4 had a significantly lower pH than the other community states at this time (*P* < .001 against each of the other community states), being similar to the pH in feces of the infants at 4 months of age (*P* > .7 to both M4 community states). This corresponds to the observed, although not significant, proximity of M12-C4 to the 4-month communities (Fig. 3A) and the greater distance of this community state to the MM samples. At 36 months, we observed that M36-C3 significantly differed from the other community states in levels of pH, acetate, butyrate, and propionate (*P* < .05), while M36-C1 and M36-C2 were not significantly different from each other in each of these measured parameters (*P* > .4). Of note, no significant correlations between fecal milieu parameters and the relative abundance of different taxa were found (Supp. Table 1).

As no clear pattern of transition between community states was observable, we aimed to predict the progression of infants between community states over time by using a multinomial logistic regression model. This included all infant samples and was trained to use the infant’s current community state as a feature and its future community state as the response variable (Fig. 3D). The low accuracy in predictability between each time point confirmed the chaotic nature of gut microbiota development. When visualized, the stochastic nature of the transitions can be clearly observed with individuals hopscotching between community states, demonstrating the complexity of the problem of predicting the future state of an infant’s microbiota (Fig. 3E).

### Differentiation of mature microbiota community states manifests around 3 years of age

**Figure 4:**
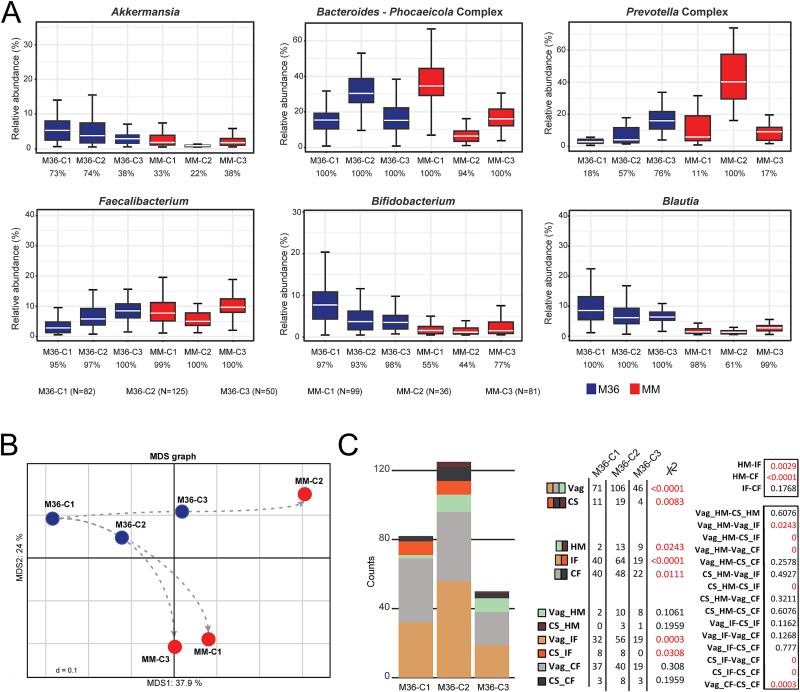
Taxonomic description of microbial community states at toddler age. A. Boxplots of the relative abundances of dominant bacterial genera showing differences between the infant microbiota community states at 36 months of age and the mature microbiota state (MM). The prevalence of each taxon within the community states is given under the name. B. MDS plot of microbiota community states at 36 months of age and adult samples. The dotted line follows a proposed trajectory of the children at 36 months of age toward the different adult community states. C. Prevalence of children within each M36 microbiota community state by birth mode and feeding group. The number of infants per community state and the *P*-values of the χ2 (Chi2) test for all the group-comparisons are displayed on the right. Abbreviations: C, community states; M, month; M4, 4-month-old infants; M12, 12-month-old infants; M24, 24-month-old infants; M36, 36-month-old infants; Vag, vaginal birth mode. Significance differences for all plots can be found in Supplementary Table 1.

Based on the observation that the infant gut microbiota at M36 showed equal richness to the MM samples (*P* = .2608; Fig. 1B), we hypothesized that the microbial community states in this age group might develop into predictable adult communities. To determine this, we identified genera that showed consistent patterns between the community states at M36 and MM (Supplementary Table 3, Fig. 4A). Given the recent reclassification of the genus *Prevotella* into seven genera based on their genomic similarity, yet the lack of corresponding complexity in their 16S rRNA gene sequences, we refer to all species within the genera *Prevotella*, *Hallella*, *Segatella*, *Hoylesella*, *Leyella*, *Xylanibacter*, and *Palleniella* as the “*Prevotella* complex” [31]. Similarly, the term “*Bacteroides-Phocaeicola* complex” is used to refer to these two genera, which remain grouped within the SILVA database. In both M36-C3 and MM-C2, we observed a lower prevalence and relative abundance of *Akkermansia* and lower relative abundance of the *Bacteroides-Phocaeicola* complex, yet higher relative abundance and prevalence of the *Prevotella* complex. These similarities led us to propose the trajectory of maturation from M36-C3 to MM-C2. This differed from the higher relative abundance of the *Bacteroides-Phocaeicola* complex seen in M36-C2 and MM-C1, and to a lesser extent in MM-C3.

Given the observed patterns in these key genera, we propose that M36-C1 is an immature community state for this time point, which can develop into either a M36-C2- or M36-C3-like community at a later time point (Fig. 4B). The suggestion of M36-C1 as an immature community was based on the relative abundance of taxa indicative of an adult microbiota. Compared to both other community states at 36 months, and to the three adult states, M36-C1 had a lower relative abundance of *Faecalibacterium* (*P* < .001), a common genus of butyrate-producing bacteria within the adult gut (Fig. 4A). Conversely, nearly all 36-month community states contained both *Blautia* and *Bifidobacterium*, yet their prevalence and relative abundance was decreased within the adult states (*P* < .001).

While we found that early life events had no significant effect on the gut microbiota at the age of 3 years (Fig. 1D), when refined to the level of community states, the distribution of samples was influenced by both delivery method and early life diet (Fig. 4C). Interestingly, even at the age of 3 years, HM-fed infants showed a preference for M36-C2 and M36-C3, proposed to be the more mature community states, with only few HM-fed infants being allocated to M36-C1 (8%), the assumed immature community state. On the other hand, the formula-fed infants showed a stronger preference for M36-C1 and M36-C2, with the IF-fed infants predominantly occurring within M36-C2 (52%). This may suggest that while the impact of early-life diet on the microbiota is mitigated by 36 months on the individual, minor effects can still be observed across a population.

## Discussion

The first years of life are very important for the development of gut microbial communities, but previous studies have suggested contradictory results regarding the trajectories of microbiome maturation. Moreover, factors such as diet and environment are known to modulate the gut microbiome of infants; however the point at which their influence wanes has rarely been determined [6], in part due to many studies focusing on the first year of life [32–35]. The sampling conducted within this project enabled investigating the effects of nutritional intervention and other early life factors on the gut microbiota development of infants up to toddler age.

We showed that the microbiota profiles of infants converged toward those of adult samples. Hereby, the most dramatic shift in distance, both phylogenetically and in terms of predicted function, occurred between 4 and 12 months. This confirms previous results that the first year of life is the most turbulent for the infant gut, as the microbial community is initially colonized and the infants’ diets change from breastfeeding to the introduction of solid foods [36]. Infant stool SCFA profiles showed decreased acetate levels while butyrate levels increased to reach a ratio of approximately 3:1:1 (acetate, propionate, butyrate) at 36 months of age, resembling values commonly known in adults. High acetate levels early in life with a decrease over time, as observed in our study, agrees with several studies from the literature and the dominance of acetate-producing *Bifidobacterium* [37–39]. Other studies have reported increasing acetate levels over time, probably due to different sampling and storage strategies, measurement techniques and cohort size [40, 41].

The microbiota was most significantly impacted by external factors (diet, mode of birth, etc.) during the first year of life. Interestingly, diet and whether an infant has siblings, had a longer lasting effect on the structure of the microbiota than the mode of birth. While no direct effect of early-life-events (mode of birth, diet) on the gut microbiota was observed by the age of 3 years, consistent with a report from the Finnish HELMi cohort and TEDDY study [8, 42], the variability of the microbiota between individuals made it difficult to identify specific differences. To account for this variability, we applied time-point-specific *de-novo* clustering of the samples to refine the analysis of community states formed during the maturation of the infant gut microbiota. By studying the community states of each infant’s gut microbiota, we determined that the diet an infant received in early life still altered the community state an infant was likely to have at 3 years of age. We identified the M36-C1 community to be immature, characterized by low levels of multiple genera and families considered markers of maturity. For example, compared to both the other community states at 36 months and the three MM communities, M36-C1 had a significantly lower relative abundance of *Faecalibacterium*, a common genus of butyrate-producing bacteria within the adult gut, consistent with observations by others [32, 43]. In contrast, M36-C1 had a significantly higher relative abundance of *Bifidobacterium*, affirming its assignment as a less mature microbiota state. These results support previous research suggesting that *Bifidobacterium* dominates the infant gut and decreases during development [44, 45].

Prior research has shown that an infant’s gut microbiota develops through a deterministic set of structures. While we observed a similar pattern overall, studying the transition of infants between community states identified a diverse range of transitions that could not be predicted from one time point to the next. This stochastic hopscotching of an individual’s microbiota across community states is likely driven by a complex set of factors [6, 33, 46]. While previous results have shown the microbiome trajectory is more predictable during the first months of life [27, 46], e.g., after 6 months of age, the current community cannot be predicted based on the previous community composition. Our findings corroborate previous results of chaotic transitioning between microbiota states up to 31 weeks of age [7] and results from the TEDDY study, which also showed a wide range of potential community states at each time point [8]. Based on these results, along with those of this study, we suggest that the infant microbiome consistently moves toward a more mature adult microbial composition, but the intermediate states after 6 months of age cannot be predicted.

This work provides greater insight into community development in early life by applying a time point specific clustering approach to longitudinal sampling of a large cohort over the first 3 years of life. However, while we propose potential trajectories that the infant microbiota may follow to mature states, further time points would be required to validate this and to understand whether established communities remain stable, or individuals continue to hopscotch between community states. This would be further facilitated by the implementation of large multicenter cohorts from different continents that harmonize protocols for detailed longitudinal sampling, high-quality metadata, and functional multi-omic methods.

## Supplementary Material

2024-11-19_Suppl-Fig-S1_ycaf016

Supplementary_Table_1_ycaf016

Supplementary_Table_2_ycaf016

Supplementary_Table_3_ycaf016

Supplementary_Table_4_ycaf016

Supplementary_Table_5_ycaf016

2025-01-30_ISMECOMMUN-D-24-00050_R2_suppl_ycaf016

## Data Availability

The raw data (16S rRNA gene amplicons) from adult reference samples are publicly available at the European Nucleotide Archive under accession PRJEB47555. The infant data is available at accession PRJEB47935.
