# Supplementary figures and images for: The infant microbiota hopscotches between community states toward maturation—longitudinal stool parameters and microbiota development in a cohort of European toddlers
Source: ISME Commun. 2025 Mar 11;5(1):ycaf016. doi: 10.1093/ismeco/ycaf016 (PMC11905755; doi:10.1093/ismeco/ycaf016)

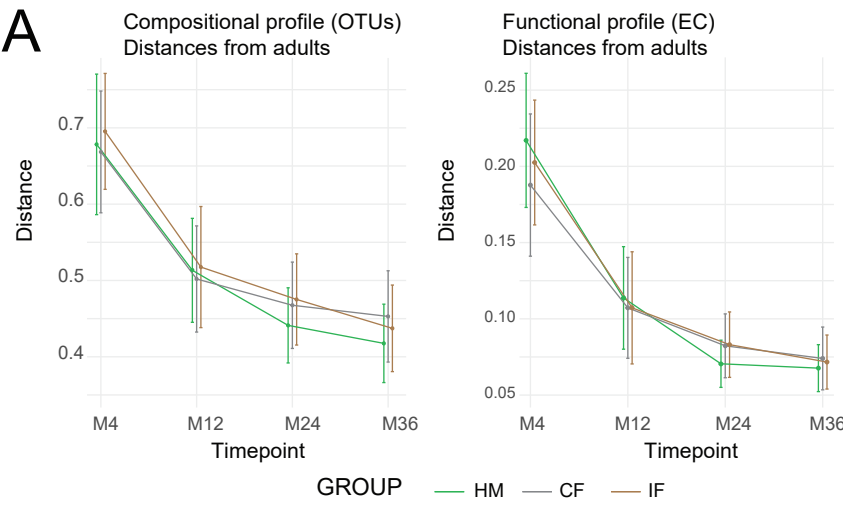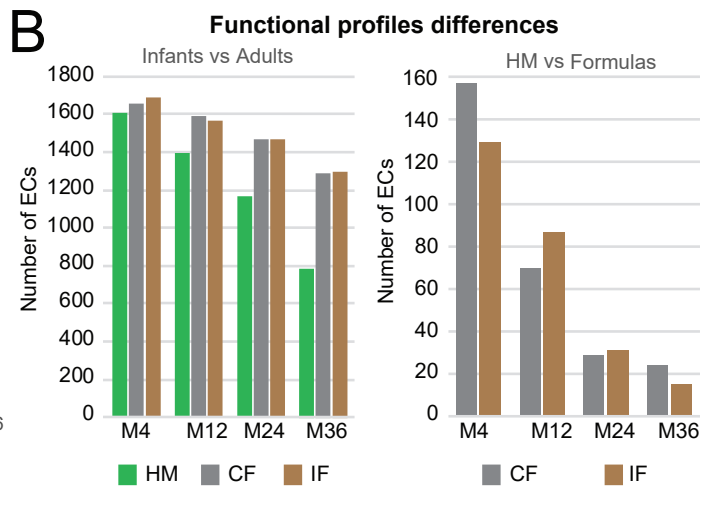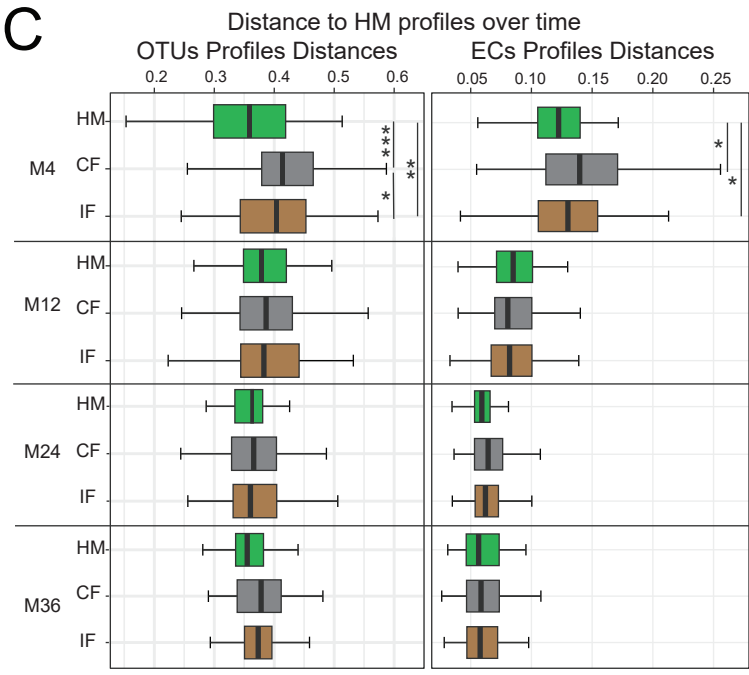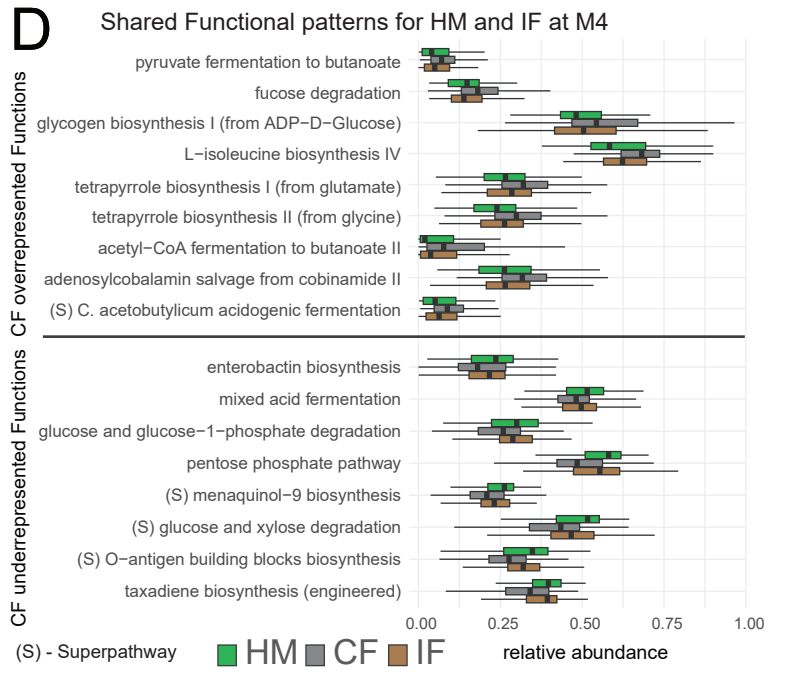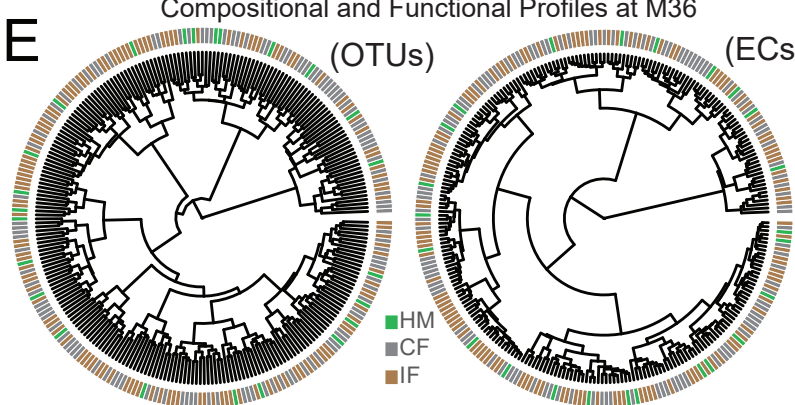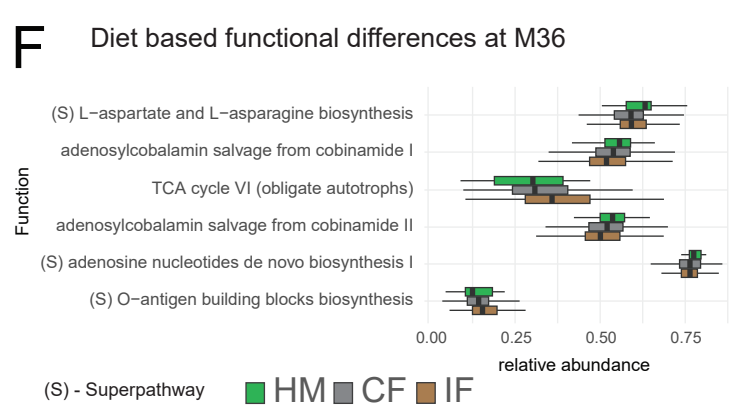

Supplement: 2024-11-19_Suppl-Fig-S1_ycaf016 [file 2024-11-19_suppl-fig-s1_ycaf016.pdf]
