# Supplementary material for: The infant microbiota hopscotches between community states toward maturation—longitudinal stool parameters and microbiota development in a cohort of European toddlers
Source: ISME Commun. 2025 Mar 11;5(1):ycaf016. doi: 10.1093/ismeco/ycaf016 (PMC11905755; doi:10.1093/ismeco/ycaf016)
Supplement: 2025-01-30_ISMECOMMUN-D-24-00050_R2_suppl_ycaf016 [file 2025-01-30_ismecommun-d-24-00050_r2_suppl_ycaf016.pdf]

The infant microbiota hopscotches between community states towards maturation – Longitudinal stool parameters and microbiota development in a cohort of European toddlers

Evangelia Intze *et al.*

Supplementary Information

To explore the effects of diet at 36 months, the taxonomic changes as presented in Fig. 2 were complemented by inferred functional data (PICRUSt2 analysis).

Similar to the taxonomic data, we observed that all three diet groups displayed the same trajectory of maturation towards profiles in the MM samples based on the functional prediction (**Supplementary Fig. S1A**; right plot). The calculated number of enzymes (ECs) was different from the MM samples and each infant time point (**Supplementary Fig. S1B**). The HM group had the lowest number of significantly different ECs to MM samples profiles at all four time points, while both formula groups were similar (Supplementary Fig. 2B, left plot). To separate the two formulas, we compared their functionality to the HM group and calculated the number of different ECs (**Supplementary Fig. S1B**, right plot). Whilst the scale of differences between the formulas and HM group was ten-fold lower than the infants to MM samples, we observed differences in the number of ECs at each time point: at 4 months, IF was more similar to HM, however, by 12 months this was reversed and after then the number of ECs different to HM were below 40 for both formulas.

Expanding upon the comparison to HM infants (reference group), we looked at the taxonomic, and functional profile distances for each of the formulas at each time point (**Supplementary Fig. S1C**). While significant differences (both taxonomically and functionally) were observed between both formulas and the HM group at 4 months, no differences were observed after this time point. Interestingly, we also observed a significant difference between the two formulas at 4 months taxonomically, with the IF group showing a slightly reduced distance to the HM group compared to CF. These results are consistent with our previously published analysis of this cohort up to M24 (1). To expand upon this, we aimed to see if these differences were reflected functionally. We identified pathways which were either over- or underrepresented in CF compared to both IF and HM groups ($p < 0.05$ between CF and both HM and IF, $p > 0.05$ between IF and HM). In total, 30 pathways were overrepresented and a further 29 underrepresented. These included multiple metabolic pathways, a selection of which are shown (**Supplementary Fig. S1D; Supplementary Table 3**); the full list is available in Supplementary Table 2. These results show that functional differences occur between dietary groups at 4 months, including *e.g*. the early production of butyrate within the CF infants, indicating an accelerated shift from HM reference profiles at this age.

While we observed that in comparison to both, the MM samples and to each other (*i.e.*, IF *vs*. CF), the dietary groups showed minor differences taxonomically, but not functionally at 36 months, we wished to validate this further. Dendrograms of the similarity of all 36-month samples to each other were generated using either the taxonomic or functional distances, and both showed no clear clustering based on the

early dietary interventions (**Supplementary Fig. S1E**). We identified 19 pathways that were differentially represented between dietary groups at 36 months. However, only six of these pathways had an average relative representation >0.1% and the differences between the feeding groups were subtle (**Supplementary Fig. S1F**, **Supplementary Table 4**).

These results suggest that diet in the first 6 months of life influences the microbiota during this period, however, the long-term impact of these changes on the microbiota as detected by amplicon sequencing are minimal.

References

1. Lagkouvardos I, Intze E, Schaubeck M, Rooney JPK, Hecht C, Piloquet H, et al. Early life gut microbiota profiles linked to synbiotic formula effects: a randomized clinical trial in European infants. The American Journal of Clinical Nutrition. 2023;117(2):326-39.

Supplementary Figure Legends

Supplementary Figure S1: Influence of diet on community states over time. A. The distance of feeding groups from the MM samples for each time point are shown at the level of microbial composition (left; GUniFrac Distance) or functional profile (right; Bray-Curtis dissimilarity). B. Number of ECs (Enzyme Commission) significantly different between the diet groups and the MM samples at each time point (left) and from the formula fed infants compared to the HM group at each time point (right). The landscape of detected ECs at each time point was determined to be consistent (M4: 1,860, M12: 1,862, M2: 1,861, M36: 1,859) with almost all potential ECs (n = 1,863) being detected at each time point. C. Boxplots of the distances between the diet groups (CF and IF) and the HM group within each time point either via microbial composition (left) or functional profile (right). D. Functional pathways at M4 that were significantly different between the HM and CF groups ($p < 0.05$), but consistent between HM and IF ($p > 0.05$). E. Cladogram of individual microbial profiles based on generalized UniFrac distances (left) or functional profiles dissimilarities using Bray-Curtis distances (right). F. Functional pathways that showed a significant ($p < 0.05$) difference between the dietary groups at M36. Abbreviations: CF, control formula; EC, enzyme commission numbers; HM, human milk; IF, intervention formula (with synbiotics); I, operational taxonomic unit. Significance differences for all plots can be found in **Supplementary Table 1**.

Supplementary Table Legends

Supplementary Table 1: P-values for all statistical tests included within this manuscript. P-values are separated based on the figure they belong to, and correlations are separated based on the time-point, and grouping (OTU or taxonomic group) being correlated.

Supplementary Table 2: Correlation of taxonomic groups with faecal parameters.

Supplementary Table 3: Significantly differentially represented pathways between dietary groups at 4 months.

Supplementary Table 4: Significantly differentially represented pathways between dietary groups at 36 months.

Supplementary Table 5: The mean relative abundance and standard deviation of each family within each community state, across all time points.
